# The First Differentiated TB Care Model From India: Delays and Predictors of Losses in the Care Cascade

**DOI:** 10.9745/GHSP-D-22-00505

**Published:** 2023-04-28

**Authors:** Hemant Deepak Shewade, Asha Frederick, G. Kiruthika, Madhanraj Kalyanasundaram, Joshua Chadwick, T. Daniel Rajasekar, K. Gayathri, R. Vijayaprabha, R. Sabarinathan, Jeyashree Kathiresan, P.K. Bhavani, S. Aarthi, K.V. Suma, Delphina Peter Pathinathan, Raghavan Parthasarathy, M. Bhavani Nivetha, Jerome G. Thampi, Deiveegan Chidambaram, Tarun Bhatnagar, S. Lokesh, Shanmugasundaram Devika, Timothy S. Laux, Stalin Viswanathan, R. Sridhar, K. Krishnamoorthy, M. Sakthivel, S. Karunakaran, S. Rajkumar, M. Ramachandran, K.D. Kanagaraj, M. Kaleeswari, V.P. Durai, R. Saravanan, A. Sugantha, S. Zufire Hassan Mohamed Khan, P. Sangeetha, R. Vasudevan, R. Nedunchezhian, M. Sankari, N. Jeevanandam, S. Ganapathy, V. Rajasekaran, T. Mathavi, A.R. Rajaprakash, Lakshmi Murali, U. Pugal, K. Sundaralingam, S. Savithri, S. Vellasamy, D. Dheenadayal, P. Ashok, K. Jayasree, R. Sudhakar, K.P. Rajan, N. Tharageshwari, D. Chokkalingam, S.M. Anandrajkumar, T.S. Selvavinayagam, C. Padmapriyadarshini, Ranjani Ramachandran, Manoj V. Murhekar

**Affiliations:** a Indian Council of Medical Research, National Institute of Epidemiology, Chennai, India.; b State TB Cell, Government of Tamil Nadu, Chennai, India.; c Indian Council of Medical Research, National Institute for Research in Tuberculosis, Chennai, India.; d World Health Organization Country Office for India, New Delhi, India.; e Tsehootsooi Medical Center, Fort Defiance, AZ, USA.; f Jawaharlal Institute of Postgraduate Medical Education and Research, Puducherry, India.; g Government Hospital of Thoracic Medicine, Tambaram, India.; h Tirunelveli Medical College Hospital, Tirunelveli, India.; i Directorate of Medical and Rural Health Services, Government of Tamil Nadu, Chennai, India.; j Directorate of Public Health and Preventive Medicine, Government of Tamil Nadu, Chennai, India.

## Abstract

To reduce TB deaths in resource-limited settings, a differentiated care strategy can be used to triage patients with high risk of severe illness (i.e., those with very severe undernutrition, respiratory insufficiency, or inability to stand without support) at diagnosis and refer them for comprehensive assessment and inpatient care. Globally, there are few examples of implementing this type of strategy in routine program settings. Beginning in April 2022, the Indian state of Tamil Nadu implemented a differentiated care strategy called Tamil Nadu-*Kasanoi Erappila Thittam* (TN-KET) for all adults aged 15 years and older with drug-susceptible TB notified by public facilities. Before evaluating the impact on TB deaths, we sought to understand the retention and delays in the care cascade as well as predictors of losses. During April–June 2022, 14,961 TB patients were notified and 11,599 (78%) were triaged. Of those triaged, 1,509 (13%) were at high risk of severe illness; of these, 1,128 (75%) were comprehensively assessed at a nodal inpatient care facility. Of 993 confirmed as severely ill, 909 (92%) were admitted, with 8% unfavorable admission outcomes (4% deaths). Median admission duration was 4 days. From diagnosis, the median delay in triaging and admission of severely ill patients was 1 day each. Likelihood of triaging decreased for people with extrapulmonary TB, those diagnosed in high-notification districts or teaching hospitals, and those transferred out of district. Predictors of not being comprehensively assessed included: aged 25–34 years, able to stand without support, and diagnosis at a primary or secondary-level facility. Inability to stand without support was a predictor of unfavorable admission outcomes. To conclude, the first quarter of implementation suggests that TN-KET was feasible to implement but could be improved by addressing predictors of losses in the care cascade and increasing admission duration.

## Abstract (precis)

The authors present the first published experience of a statewide differentiated TB care model from Tamil Nadu, India. The findings of this operational research guided the authors in closing the gaps in the care cascade.

## Teaser

**Key Findings:**

- To reduce TB deaths in resource-limited settings, triaging at diagnosis can identify those with immediate need for comprehensive assessment and inpatient care.
- This type of differentiated TB care model was successfully implemented in Tamil Nadu, India, without additional stress on the health system.
- Half of the patients referred as a result of triaging were very severely undernourished, which implies a need for capacity-building of inpatient care facilities in clinical management of very severe undernutrition in adults.
- The identification of predictors of not being triaged and comprehensively assessed will inform ongoing and future improvements to the care model.

**Key Implications:**

- The state should immediately focus on increasing the duration and quality of inpatient care, particularly for very severe undernutrition.
- Specific strategies should be developed to address the predictors of not being triaged and not being comprehensively assessed.

## INTRODUCTION

Due to the increase in estimated TB deaths, COVID-19 is a potential setback to India’s ambitious plan to attain the World Health Organization’s End TB Strategy targets for the year 2030 by 2025.^1^^–^^3^ India has the highest TB burden with an estimated case fatality ratio of 19:100 in 2020 (increased from 17:100 in 2019, 7.3% higher when compared to 2015).^3^ The solutions to addressing high mortality range from ramping up TB case finding and systematic assessment of severe illness at diagnosis followed by appropriate care to enhanced food rations (including pulses) and direct cash transfers to the poor pending restoration of livelihoods.^4^

In January 2021, India’s National TB Elimination Program (NTEP) recommended severity assessment for all notified patients at diagnosis and referral of severely ill patients for inpatient care (Table 1). This differentiated TB care guidance also recommended modifications that considered the local context and public health capacity.^5^

**TABLE 1.** Scoring Based on Comprehensive Assessment and Criteria for Patients Requiring Inpatient Care^a^ Among Adults With TB^b^

**Criteria**	**Considered to Be Emergency** ^c^	**Scoring Criteria**	**Score Assigned** ^d^
Pulse rate per minute		<60	2
		>100 (persistent after 30 minutes)	2
BMI, kg/m^2^	Yes (<14)	<14	1
		<16 with pedal edema	1
		>40	1
MUAC, cm		<16	1
Temperature, Celsius	Yes (<35, >41)	<35	2
		>41	2
Blood pressure, mmHg	Yes	Hypertension (≥140/90)	2
		Hypotension (diastolic <60)	2
Respiratory rate, breaths/minute	Yes (>24)	18–24	1
		25–30	2
		>30	3
Oxygen saturation, %	Yes (<94)	90–93	1
		85–89	2
		<85	3
Hemoglobin, g/dL	Yes (<7)	<7	2
Icterus		Present	1
Pedal edema		Present	1
General condition	Yes (if unable to walk, drowsy, unconscious)	Inability to walk but conscious and oriented	1
		Conscious, not oriented	2
		Drowsy	3
HIV		Positive and on ART	1
		Positive and not on ART	2
Random blood sugar, mg/dL		<70	2
		>200	2
Total WBC count, WBC/microliter		Total count >11,000	1
		Total count <4,000	1
Chest radiograph	Yes (massive pneumothorax, hydropneumothorax)	Hydropneumothorax	3
		Bilateral consolidation	2
Hemoptysis	Yes	Present	3

Abbreviations: ART, antiretroviral therapy; BMI, body mass index; MUAC, mid-upper arm circumference; WBC, white blood cell.

a Modifications to the criteria provided by Central TB Division 2021 technical guidance^5^: blood sugar of >128 replaced with >200. In addition, inpatient care may be provided irrespective of the total score or presence/absence of emergency criteria if the treating physician feels the need for inpatient care.

b Based on Central TB Division 2021 Technical Guidance on Differentiated Care of TB Patients in India.^5^

c If there is an indicator suggesting emergency, the inpatient care should be provided irrespective of the total score.

d Patients with total score more than 1 to be provided inpatient care, and patients with total score more than 3 to be provided inpatient care in a facility with intensive care unit.

There were 2 challenges to meeting these recommendations. First, NTEP did not specify the exact modality of implementation, including standard operating procedures and the framework to be used for recording, reporting, monitoring, and evaluation. Second, the criteria in the guidance to assess severe illness included 16 indicators (Table 1). Therefore, assessment could be time-consuming and require clinical and diagnostic capacity not uniformly available in the facilities that diagnose and treat TB (peripheral health institutions [PHI]), particularly those in the periphery.^5^ As most TB deaths occur within 2 months (early deaths) and half of early deaths happen within the first 2 weeks of diagnosis,^6^ assessment mandates a quick decision at diagnosis.

Hence, a potentially feasible strategy is to introduce a triage tool for use in all PHIs irrespective of their clinical and diagnostic capacity (Box, Supplement 1).^7^^–^^9^ Using the 5 indicators in this tool, patients with very severe undernutrition, respiratory insufficiency, or poor performance status (also known as high risk of severe illness, [Box]) may be identified at diagnosis and prioritized for comprehensive assessment and inpatient care.^7^ Past pilots from routine program settings in India suggested that the tool was feasible for use even by paramedical staff.^8^^,^^9^ Early TB deaths were observed in 14% of those with very severe undernutrition and 18% of those with poor performance status at diagnosis.^6^ However, these pilots did not systematically link triaged patients for appropriate care.^8^^,^^9^

BOX Triage Tool for Severe Illness at Diagnosis Among Adults^a^ With TB Without Known Drug-Resistance^b^

If at least 1 of the following is present, then the person with TB is at high risk of severe illness (requires referral for comprehensive assessment, confirmation of severe illness, and inpatient care):

1. Body mass index (BMI) less than or equal to (≤) 14.0 kg/m^2c^
2. BMI less than or equal to (≤) 16.0 kg/m^2^ with leg swelling^c^
3. Respiratory rate more than (>) 24 breaths/minute^d^
4. Oxygen saturation less than (<) 94%^d^
5. Not able to stand without support (poor performance status—standing with support/squatting/sitting/bed ridden)

^a^ Aged 15 years and older.

^b^ Reprinted from Shewade et al.^8^ under a CC BY license, with permission from MDPI, © MDPI 2021; tool adapted from Bhargava et al.^7^

^c^ Very severe undernutrition indicators.

^d^ Respiratory insufficiency indicators.

Starting April 2022, with the goal to reduce deaths by 30% among adults aged 15 years and older with TB notified by public facilities (public PHIs), the state of Tamil Nadu in southern India implemented Tamil Nadu-*Kasanoi Erappila Thittam* (TN-KET), meaning “TB death free project” in Tamil. Statewide in routine program settings, we triaged people with TB at diagnosis (Box), followed by referral for comprehensive assessment, confirmation of severe illness (Table 1), and inpatient care.^10^

Globally and nationally, there are limited examples of implementing such a strategy in routine TB program settings. Before evaluating the impact on TB deaths, it was crucial to understand the retention and delays in the TN-KET care cascade, as well as predictors of losses (especially during the early stages of implementation). Hence, in this operational research (April–June 2022), we assessed the (1) burden of high risk of severe illness; (2) feasibility of implementing TN-KET (retention and delay in care cascade from TB diagnosis to discharge for ambulatory directly observed treatment); and (3) predictors of not being triaged, not being comprehensively assessed, and unfavorable admission outcomes.

We sought to understand the retention and delays in the TN-KET care cascade as well as predictors of losses.

## METHODS

### Study Design

This was a cohort study involving secondary program data. For predictors of not being triaged and burden of high risk of severe illness, we followed a cross-sectional design.

### Setting

Tamil Nadu has a population of about 72 million. In 2021, Tamil Nadu was ranked second among all large states based on a health index developed by the premier policy think tank of the Government of India.^11^ The proportion of undernutrition (body mass index [BMI]<18.5 kg/m^2^) among women (aged 15–49 years) in the general population is 12.6%, while it is 12.1% among men (aged 15–49 years).^12^

Tamil Nadu’s TB case notification rate (reported data) in 2021 was 100 per 100,000 population (approximately 4,500 public notifications per month).^13^ The treatment success rate of the 2020 cohort was 82%. The case fatality increased from 3.4% in 2019 to 6.4% in 2020.^13^^,^^14^

Tamil Nadu’s NTEP infrastructure includes 35 districts (30 excluding Chennai, the capital city), 461 subdistrict-level administrative TB units, and 5,253 PHIs (2,788 public PHIs). A district program coordinator supports the district TB officer. In the public sector, the lowest level of PHI is the primary health center, which has at least 1 medical doctor and a designated microscopy center, each with a laboratory technician for sputum microscopy.

Paper-based registers maintained at PHIs and TB units have a list of patients notified, their management, and the treatment outcomes. Each TB unit has a senior TB treatment supervisor (STS) who updates these details from the register in NIKSHAY (a case-based, web-based electronic TB information management system) using their mobile tablet.^15^ Patients receive daily ambulatory directly observed treatment by a health care provider, community volunteer, or a family member.

### Study Population

We included all adults aged 15 years and older with TB (not known to be drug-resistant at diagnosis) notified during April–June 2022 from all the public PHIs of the 30 NTEP districts in Tamil Nadu (except Chennai). We included patients irrespective of their treatment initiation or transfer-out status. We excluded patients transferred in from other non-study districts.

We did not include children aged younger than 15 years with TB and patients known to be drug-resistant at diagnosis because children are diagnosed by a pediatrician at a higher-level facility, while the patients with drug-resistant TB undergo mandatory comprehensive baseline assessment at district-level centers for drug-resistant TB.

### TN-KET Care Cascade

TN-KET is an initiative of the state health system with the involvement of all 3 key directorates.^10^ The Directorate of Medical Education provides tertiary-level care through teaching hospitals (1 per district). The Directorate of Medical and Rural Health Services provides secondary-level care through district headquarter and subdistrict hospitals. The Directorate of Public Health and Preventive Medicine provides primary-level care through primary health centers. Between December 2021 and February 2022, we completed the preparation of tools, standard operating procedures, monitoring mechanisms, and trainings. After a 2-week statewide pilot in March 2022, implementation began in April 2022.^10^

#### Triaging

We introduced a paper-based triage tool (to be used at TB diagnosis) in all the public PHIs (Box, Supplement 1).^10^ Triaging was done by health staff at the public PHI, which could include the laboratory technician, TB health visitor (if present), STS (if based at the public PHI), staff nurse, or the medical officer. Even on their own, very severe undernutrition, respiratory insufficiency, and poor performance status are known risk factors for death and have a strong association with TB mortality.^16^^–^^24^

#### Referral and Appropriate Care

For people with high risk of severe illness, the diagnosing TB unit STS facilitated the referral (using public ambulance service) to nodal TN-KET inpatient care facilities for comprehensive assessment, confirmation of severe illness, and inpatient care.^10^ Table 1 contains the criteria for confirmation of severe illness after comprehensive assessment.^5^

Tamil Nadu earmarked roughly 150 nodal TN-KET inpatient care facilities and about 900 TB beds (10% being in high-dependency units). Within a district, this included teaching hospitals, the district headquarters hospital, and select subdistrict hospital(s) with a chest or internal medicine physician.^10^ The State TB Cell of Tamil Nadu prepared the *Inpatient Care Guide for Adults With TB Who Are Severely Ill* with clinical guidance to assist the nodal physicians.^10^^,^^25^ This includes management of TB complications, management of comorbidities, and discharge criteria.^10^^,^^25^

### Data Capture

At the time of TB notification, the STS transcribed the triaging details in the Severe TB Web Application (TB SeWA), a closed-access web app that allowed mobile or tablet-based data capture.^10^^,^^26^ For the STS to cross-check the interpretation in the paper-based triage tool, TB SeWA autogenerated BMI and high risk of severe illness status. Later, if applicable, the STS captured post-referral (referral [yes/no], comprehensive assessment [yes/no], and confirmation of severe illness [yes/no]) and post-admission details (admission for inpatient care [yes/no], issue to be addressed during inpatient care, and discharge outcome).

### Coordination and Monitoring

For better coordination between PHIs, the district NTEP team, and nodal inpatient facilities, stakeholder contact details for all 30 districts were shared in a cloud-based online monitoring tool. Under the guidance of the district TB officer, at least once per week and at the end of the month/quarter, the district program coordinator monitored the gaps in the care cascade, provided feedback for corrective actions, and indicated this in the online monitoring tool (yes/no). Through TB SeWA, districts had access to aggregate and individual-level TN-KET care cascade data.^10^ We have summarized the TN-KET care cascade and the corresponding monitoring indicators in the Figure and the standard operating procedure in Supplement 2. Details of how TN-KET was implemented in the form of “11 tips” have been published elsewhere.^10^

### Data Management and Statistics

On July 29, 2022, we merged NIKSHAY data with TB SeWA data using NIKSHAY identifier (NIKSHAY was the parent database). Supplement 3 contains the anonymized data in MS Excel format. We analyzed the data using Stata 16.1 (StataCorp) software.

We used proportions to document the retention in the TN-KET care cascade (Figure). Among those triaged, we calculated the delays, completeness of information on 5 indicators, and burden of high risk of severe illness. We calculated median (interquartile range [IQR]) admission duration in days among admitted confirmed severely ill patients who were discharged for ambulatory directly observed treatment. We identified the number of districts that met the “’80-80-80-7” target, a realistically achievable arbitrary target for our monitoring indicators (80% triaged, 80% comprehensive assessment among those with high risk of severe illness, 80% admission of confirmed severely ill, and median 7 days admission duration).

**FIGURE.**
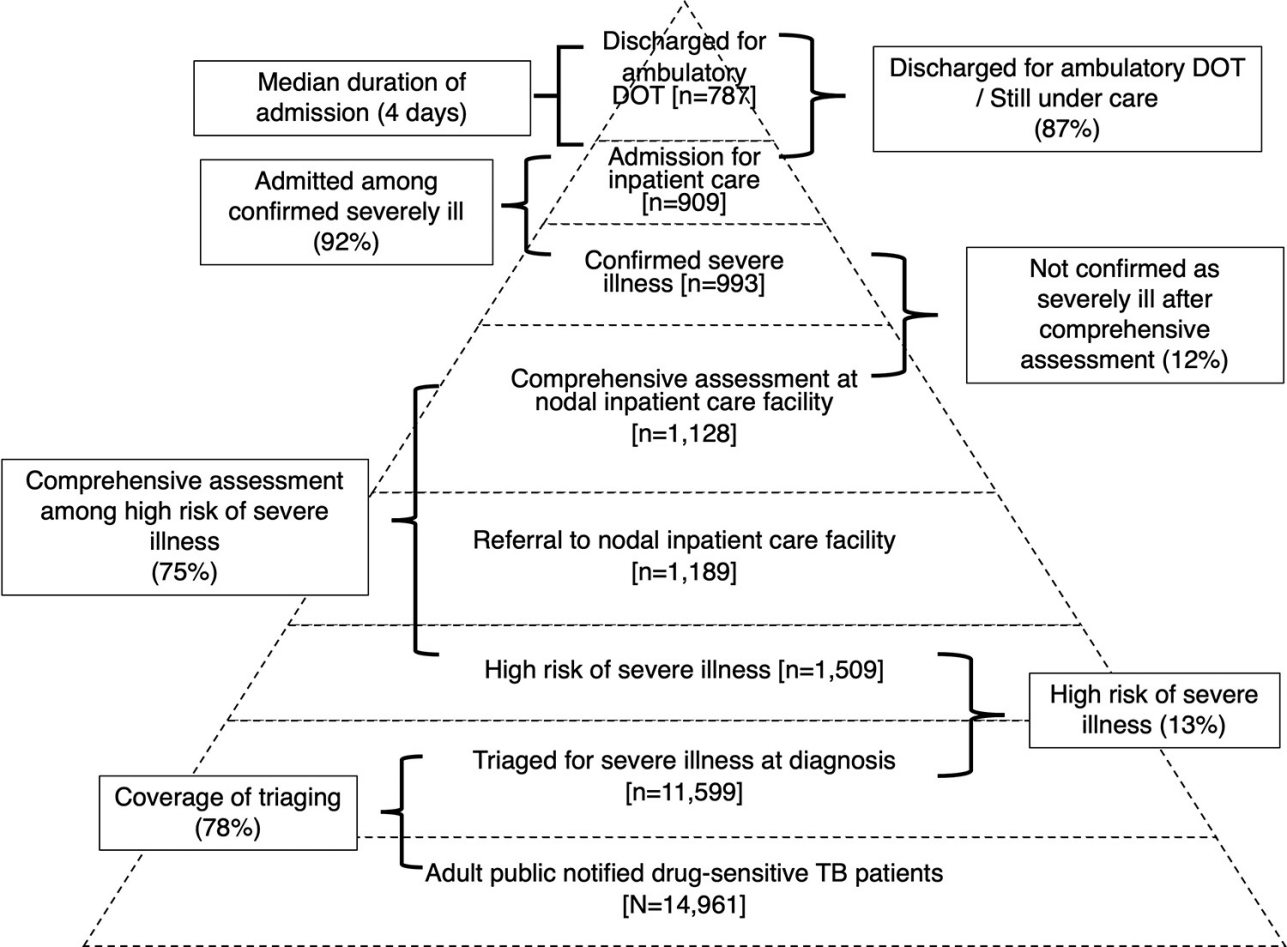
TN-KET Care Cascade^a^ at Diagnosis Among Adults^b^ With TB Without Known Drug-Resistant Disease That Were Notified by Public Facilities^c^ in Tamil Nadu, India, April–June 2022 Abbreviations: TN-KET, Tamil Nadu-*Kasanoi Erappila Thittam* (Tamil Nadu “TB death free program”); DOT, directly observed treatment. ^a^ As of July 29, 2022. ^b^ Aged 15 years and older. ^c^ In the 30 districts of Tamil Nadu, there were about 2,800 facilities that diagnosed TB; 150 nodal inpatient care facilities and 900 TB beds were identified.

We used modified Poisson regression with robust variance estimates to identify the predictors for not being triaged, not being comprehensively assessed, and unfavorable admission outcomes (leave against medical advice, referred to other facility, and outcome unknown/death combined). We summarized the association using adjusted prevalence ratios (aPR) for predictors of not being triaged and adjusted relative risk (aRR) for predictors of not being comprehensively assessed and unfavorable admission outcomes. We included factors with crude *P*<.05 in the multivariable regression model after ruling out multicollinearity and considering the variation in coverage of tri-aging across the districts.

### Ethical Approval

We received ethical approval from the Institutional Human Ethics Committee of the Indian Council of Medical Research-National Institute of Epidemiology (ICMR-NIE), Chennai, India (NIE/IHEC/202201-09). We used verbal consent (rather than written consent) to mimic the implementation in routine program settings and enable assessment of feasibility, which was approved by the ethics committee. We obtained the necessary administrative approvals.

## RESULTS

### Baseline Characteristics

Of the 14,961 adults aged 15 years and older with TB diagnosed/notified, the number notified per district varied from 40 to 1,336. Of 14,961 people, 2,778 (19%) were diagnosed through rapid molecular tests, 2,372 (16%) were diagnosed at primary-level PHIs, and 1,987 (13%) were transferred out of the district (Table 2).

**TABLE 2.** Predictors of Not Being Triaged^a^ for Severe Illness at Diagnosis Among Adults^b^ With TB Without Known Drug-Resistant Disease That Were Notified by Public Facilities, Tamil Nadu, India, April–June 2022^c^

	**Total, No. (%)** ^d^	**Not Triaged,**^a^ **No. (%)**^e^	**PR** **(95% CI)**	**aPR**^f^ **(95% CI)**
**Total**	14,961 (100)	3,362 (22.5)		
Age, years				
15–24	1,508 (10.1)	345 (22.9)	0.99 (0.88, 1.11)	0.93 (0.83, 1.04)
25–34	1,798 (12.0)	405 (22.5)	0.97 (0.87, 1.08)	0.91 (0.82, 1.02)
35–44	2,786 (18.6)	646 (23.2)	Ref	Ref
45–54	3,683 (24.6)	851 (23.1)	1.00 (0.91, 1.09)	1.02 (0.93, 1.11)
55–64	3,215 (21.5)	714 (22.2)	0.96 (0.87, 1.05)	1.00 (0.91, 1.10)
**≥**65	1,971 (13.2)	401 (20.4)	0.88 (0.79, 0.98)	0.90 (0.81, 1.00)
Gender				
Men	10,715 (71.6)	2,352 (22.0)	Ref	Ref
Women	4,241 (28.4)	1,009 (23.8)	1.08 (1.02, 1.16)	1.03 (0.96, 1.10)
Transgender	5 (0.03)	1 (20.0)	--	
Test used for diagnosis				
Rapid molecular tests	2,778 (18.6)	840 (30.2)	1.67 (1.55, 1.79)	1.47 (1.36, 1.59)^g^
Microscopy/culture	8,015 (53.6)	1,455 (18.2)	Ref	Ref
Chest radiograph	1,546 (10.3)	412 (26.7)	1.47 (1.34, 1.61)	1.24 (1.04, 1.47)^g^
Others	2,622 (17.5)	655 (25.0)	1.38 (1.27, 1.49)	0.98 (0.81, 1.19)
Microbiological confirmation				
Yes	11,333 (75.8)	2,406 (21.2)	Ref	Ref
No	3,628 (24.3)	956 (26.4)	1.24 (1.16, 1.32)	1.19 (0.99, 1.42)
Site of TB				
Pulmonary	12,046 (80.5)	2,361 (19.6)	Ref	Ref
Extrapulmonary	2,389 (16.0)	640 (26.8)	1.37 (1.27, 1.47)	1.21 (1.09, 1.36)^g^
Missing	526 (3.5)	361 (68.6)	3.50 (3.27, 3.75)	2.60 (2.36, 2.87)^g^
Previous treatment				
Yes	1,606 (10.7)	351 (21.9)	0.97 (0.88, 1.07)	N/A^h^
No	13,355 (89.3)	3,011 (22.6)	Ref	
HIV				
Positive	673 (4.5)	194 (28.8)	1.32 (1.17, 1.49)	1.06 (0.93, 1.20)
Negative	14,161 (94.7)	3,088 (21.8)	Ref	Ref
Unknown	127 (0.9)	80 (63.0)	2.89 (2.52, 3.31)	1.03 (0.86, 1.23)
DM				
Positive	3,823 (25.6)	745 (19.5)	0.88 (0.82, 0.95)	0.94 (0.87, 1.91)
Negative	10,710 (71.6)	2,374 (22.2)	Ref	Ref
Unknown	38 (0.3)	17 (44.7)	2.02 (1.41, 2.88)	1.28 (0.86, 1.91)
Missing	390 (2.6)	226 (58.0)		1.42 (1.23, 1.63)^g^
Peripheral health institute—notification facility				
Primary	2,372 (15.9)	414 (17.5)	Ref	Ref
Secondary	5,970 (39.9)	1,168 (19.6)	1.12 (1.01, 1.24)	0.98 (0.88, 1.08)
Tertiary	6,449 (43.1)	1,710 (26.5)	1.52 (1.38, 1.67)	1.11 (1.01, 1.23)^g^
Missing	170 (1.1)	70 (41.2)	2.36 (1.93, 2.88)	2.21 (1.83, 2.68)^g^
Treatment started				
Yes	14,435 (96.5)	3,001 (20.8)	Ref	N/A^i^
No	526 (3.5)	361 (68.6)	3.30 (3.09, 3.53)	
Transferred out of district				
Yes	1,987 (13.3)	670 (33.7)	1.63 (1.51, 1.74)	1.50 (1.40, 1.61)^g^
No	12,974 (86.7)	2,692 (20.8)	Ref	Ref

Abbreviations: aPR, adjusted prevalence ratio; CI, confidence interval; DM, diabetes mellitus; PR, crude prevalence ratio; SeWA, Severe TB Web Application.

a Triaged defined as collecting and syncing of severity details in a mobile application irrespective of the extent of missing data.

b Aged 15 years and older.

c Source is the routinely collected baseline data in NIKSHAY and data updated in TB SeWA as of July 29, 2022.

d Column percentage.

e Row percentage.

f Modified Poisson regression with robust variance estimation; results have been adjusted for district.

g *P*<.05.

h Variables with crude *P* value ≥.05 (Chi square test) were not included in the adjusted analysis.

i Excluded because of variance inflation factor >10.

### Burden of High Risk of Severe Illness

Among 11,599 adults triaged, the distribution of weight, BMI, respiratory rate, and oxygen saturation is shown in Table 3. Data on all 5 indicators were available for 99% of patients. Severe undernutrition (BMI<16 kg/m^2^) was found in 2,939 (25%) and very severe undernutrition (BMI<14 kg/m^2^ or <16 with leg swelling) in 729 (6.3%). Among those patients triaged, 1,509 (13%) were at high risk of severe illness (95% confidence interval [CI]=12.4, 13.6); of them, 729 (48%) were very severely undernourished, 748 (50%) had respiratory insufficiency, and 436 (29%) were unable to stand without support (Table 4). There was some overlap between criteria.

**TABLE 3.** Distribution of Body Mass Index, Respiratory Rate, and Oxygen Saturation at Diagnosis Among Adults^a^ With TB Without Known Drug-Resistant Disease at Diagnosis That Were Notified by Public Facilities, Tamil Nadu, India, April–June 2022^b^

	**Total,** **No. (%)**^c^	**Male,** **No. (%)**^c^	**Female,** **No. (%)**^c^	**Transgender,** **No. (%)**^c^
**Total**	11,599 (100.0)	8,363 (100.0)	3,232 (100.0)	4 (100.0)
Weight, kg				
<30	187 (1.6)	51 (0.6)	136 (4.2)	0 (0.0)
30–44.9	4,710 (40.6)	3,107 (37.2)	1,602 (49.6)	1 (25.0)
45–59.9	5,126 (44.2)	3,998 (47.8)	1,125 (34.8)	3 (75.0)
≥60	1,575 (13.6)	1,206 (14.4)	369 (11.4)	0 (0.0)
Missing	1 (0.01)	1 (0.01)	0 (0.0)	0 (0.0)
Mean (SD)	47.5 (10.8)	48.5 (10.5)	44.9 (11.3)	50 (5.3)
Body mass index, kg/m^2^				
≤14.0	699 (6.0)	458 (5.5)	241 (7.5)	0 (0.0)
14.1–16.0	2,240 (19.3)	1,656 (19.8)	584 (18.1)	0 (0.0)
16.1–18.4	3,119 (26.9)	2,337 (27.9)	779 (24.1)	3 (75.0)
≥18.5^d^	5,537 (47.7)	3,910 (46.8)	1,626 (50.3)	1 (25.0)
Missing	4 (0.03)	2 (0.02)	2 (0.1)	0 (0.0)
Mean (SD)	18.8 (4.0)	18.7 (3.7)	19.2 (4.4)	18.1 (1.6)
Respiratory rate, breaths per minute				
<18	1,114 (9.6)	796 (9.5)	317 (9.8)	1 (25.0)
18–24	9,796 (84.5)	7,055 (84.4)	2,738 (84.7)	3 (75.0)
25–30	445 (3.8)	339 (4.1)	106 (3.3)	0 (0.0)
>30	133 (1.2)	92 (1.1)	41 (1.3)	0 (0.0)
Missing	111 (1.0)	81 (1.0)	30 (0.9)	0 (0.0)
Oxygen saturation, %				
≥94	11,290 (97.3)	8,129 (97.2)	3,158 (97.7)	3 (75.0)
90–93	181 (1.6)	136 (1.6)	44 (1.4)	1 (25.0)
85–89	69 (0.6)	52 (0.6)	17 (0.5)	0 (0.0)
<85	44 (0.4)	34 (0.4)	10 (0.3)	0 (0.0)
Missing	15 (0.1)	12 (0.1)	3 (0.1)	0 (0.0)

Abbreviation: SD, standard deviation.

a Aged 15 years and older.

b Of 14,961 patients, a total of 11,599 (77.5%) were triaged.

c Column percentage.

d 1,274 were overweight (BMI 23.0–27.4) and 363 obese (BMI ≥27.5).

**TABLE 4.** Burden of High Risk of Severe Illness at Diagnosis Among Adults^a^ With TB Without Known Drug-Resistant Disease That Were Notified by Public Facilities, Tamil Nadu, India, April–June 2022^b^

**Criteria**	**No. (%)** **(N=11,599)**^b^	**95% CI**
Using the screening criteria	1,509 (13.0)	(12.4, 13.6)
Using BMI ≤14	699 (6.0)	(5.6, 6.5)
Using BMI 14–16 with leg swelling	30 (0.3)	(0.2, 0.4)
Using respiratory rate >24 breaths/minute	578 (5.0)	(4.6, 5.4)
Using oxygen saturation <94%	294 (2.5)	(2.3, 2.8)
Inability to stand without support	436 (3.8)	(3.4, 4.1)
Very severe undernutrition related indicator (any one)^c^	729 (6.3)	(5.9, 6.7)
Respiratory insufficiency related indicator (any one)^d^	748 (6.4)	(6.0, 6.9)

Abbreviations: BMI, body mass index (kg/m^2^); CI, confidence interval.

a Aged 15 years and older.

b Of 14,961 people with TB, a total of 11,599 (77.5%) were triaged as of July 29, 2022.

c Body mass index ≤14kg/m^2^/14.1–16.0 with bilateral pedal edema.

d Respiratory rate >24 breaths/minute or oxygen saturation <94%.

### Retention in TN-KET Care Cascade

We have depicted the retention/losses in the TN-KET care cascade in the Figure. Of 14,961 drug-susceptible TB patients notified by public facilities, 11,599 (78%) were triaged (district-wise range: 57%–94%). As the number notified from the districts increased, the coverage of triaging significantly decreased (r=−0.51; *P*=.004). Of 11,599 adults triaged, 1,509 were classified as high risk of severe illness; of them, 1,128 (75%) were comprehensively assessed. Of those comprehensively assessed, 135 (12%) were not confirmed as having severe illness.

Of 993 adults confirmed severely ill, 909 (92%) were admitted; of them, 787 (87%) were discharged for ambulatory directly observed treatment, 45 (5%) were still under care, 36 (4%) died, 27 (3%) left against medical advice, and 9 (1%) were referred elsewhere with outcome unknown. The median admission duration was 4 (IQR: 2, 7) days.

At the state level, we did not meet the 80-80-80-7 targets in this quarter. We have depicted the district-level information on meeting these targets in Table 5.

**TABLE 5.** Percentage of Districts That Achieved Indicator Level Targets Under TN-KET, Tamil Nadu, April–June 2022

**Indicator**	**Target**	**No. (%) (N=30)**
Triaged among adults with drug-sensitive TB at diagnosis notified by public facilities	80%	17 (56.7)
Comprehensively assessed among adults with high risk of severe illness	80%	18 (60.0)
Admitted among confirmed severely ill	80%	26 (86.7)
Median duration of admission	7 days	4 (13.3)
Any 2 indicator targets achieved		12 (40.0)
Any 3 indicator targets achieved		11(36.7)
All 4 indicator targets achieved		1 (3.3)
None achieved		2 (6.7)

Abbreviation: TN-KET, Tamil Nadu - *Kasanoi Erappila Thittam* (Tamil Nadu “TB death free program”).

### Delays in TN-KET Care Cascade

Of 11,599 triaged, the median time interval from diagnosis to triaging at a PHI was 1 (IQR: 0, 4) day, from triaging at a PHI to capturing details in TB SeWA was 3 (IQR: 1, 9) days, and from diagnosis to capturing details in TB SeWA was 6 (IQR: 3, 12) days. Of those confirmed as severely ill, 61% were already admitted at the time of triaging. Hence, among those admitted under TN-KET, the median time interval from diagnosis to admission was zero (IQR: 0, 2). Among those not already admitted at time of triaging, the median time interval from diagnosis to admission was 1 day (IQR: 0, 4).

### Predictors of Losses in TN-KET Care Cascade

We have depicted the predictors of not being triaged in Table 2. On adjusted analysis, the predictors were: diagnosis by rapid molecular tests or chest radiograph, diagnosis of extrapulmonary TB, diagnosis in a tertiary-level facility, and transfer out of district for treatment.

Comprehensive assessment was not significantly associated with number of notifications in the district (r=−0.33; *P*=.077). Among those with high risk of severe illness, we have depicted the proportion not comprehensively assessed, overall and stratified by triaging results, in Table 6. Those who were able to stand without support were 40% less likely to be comprehensively assessed when compared to those not able to stand without support. Other actionable predictors of not being comprehensively assessed were: aged 25–34 years and diagnosis at a primary or secondary level facility. The only significant predictor of unfavorable admission outcomes was poor performance status. Those with poor performance status at diagnosis were 46% more likely to develop an unfavorable admission outcome when compared to those without (aRR=1.46; 95% CI=1.04, 2.03) (data not shown).

**TABLE 6.** Predictors of Not Being Comprehensively Assessed Among Adults^a^ With TB (Without Known Drug-Resistant Disease at Diagnosis) with High Risk Of Severe Illness^b^ at Diagnosis That Were Notified by Public Facilities in Tamil Nadu, India, April–June 2022

**Baseline Characteristics** ^c^	**Total, No. (%)** ^d^	**Not Comprehensively** **Assessed,** **No. (%)**^e^	**RR**	**95% CI**	**aRR** ^f^	**95% CI**
**Total**	1,509 (100)	381 (25.3)				
Age, years						
15–24	144 (9.5)	37 (25.7)	1.02	(0.72, 1.44)	1.07	(0.76, 1.50)
25–34	168 (11.1)	60 (35.7)	1.41	(1.05, 1.89)	1.47	(1.10, 1.95)^g^
35–44	253 (16.8)	64 (25.3)	Ref		Ref	
45–54	359 (23.8)	97 (27.0)	1.07	(0.71, 1.40)	1.07	(0.82, 1.40)
55–64	346 (22.9)	74 (21.4)	0.85	(0.63, 1.13)	0.87	(0.65, 1.15)
**≥**65	239 (15.8)	49 (20.5)	0.81	(0.58, 1.12)	0.88	(0.63, 1.22)
Gender						
Men	1,062 (70.4)	263 (24.8)	Ref		N/A^h^	
Women	446 (29.6)	117 (26.2)	1.06	(0.88, 1.28)		
Transgender	1 (0.07)	1 (100.0)	--			
Test used for diagnosis						
Rapid molecular tests	213 (14.1)	53 (24.9)	0.99	(0.77, 1.29)		
Microscopy/culture	927 (61.4)	232 (25.0)	Ref		N/A^h^	
Chest radiograph	153 (10.1)	47 (30.7)	1.23	(0.94, 1.60)		
Others	216 (14.3)	49 (22.7)	0.91	(0.69, 1.19)		
Microbiological confirmation						
Yes	1,206 (79.9)	298 (24.7)	Ref		N/A^h^	
No	303 (20.1)	83 (27.4)	1.10	(0.90, 1.37)		
Site of TB						
Pulmonary	1,312 (86.9)	334 (25.5)	Ref		N/A^h^	
Extrapulmonary	175 (11.6)	43 (24.6)	0.97	(0.73, 1.27)		
Missing	22 (1.5)	4 (18.2)	0.71	(0.29, 1.74)		
Previous treatment						
Yes	212 (14.1)	47 (22.2)	0.86	(0.66, 1.13)		
No	1,297 (86.0)	334 (25.8)	Ref		N/A^h^	
HIV						
Positive	65 (4.3)	13 (20.0)	0.78	(0.48, 1.29)		
Negative	1,440 (95.4)	367 (25.5)	Ref		N/A^h^	
Unknown	4 (0.3)	1 (25.0)	0.98	(0.18, 5.37)		
DM						
Positive	325 (21.5)	78 (24.0)	0.93	(0.75, 1.15)		
Negative	1,158 (76.7)	300 (25.9)	Ref		N/A^h^	
Unknown	5 (0.3)	0 (0.0)	–			
Missing	21 (1.4)	3 (14.3)	0.55	(0.19, 1.58)		
Peripheral health institute—notification facility						
Primary	283 (18.8)	111 (39.2)	2.11	(1.68, 2.65)	1.90	(1.52, 2.40)^g^
Secondary	665 (44.1)	166 (25.0)	1.34	(1.08, 1.67)	1.26	(1.01, 1.57)^g^
Tertiary	555 (36.8)	103 (18.6)	Ref		Ref	
Missing	6 (0.4)	1 (16.7)	0.90	(0.15, 5.42)	0.82	(0.11, 5.88)
Very severe undernutrition^i^						
Yes	718 (47.6)	190 (26.5)	1.10	(0.92, 1.30)	N/A^h^	
No	791 (52.4)	191 (24.2)	Ref			
Respiratory insufficiency^j^						
Yes	747 (49.5)	215 (28.8)	1.32	(1.11, 1.57)	1.21	(1.01, 1.45)^g^
No	762 (50.5)	166 (21.8)	Ref		Ref	
Poor performance status (not able to stand without support)						
Yes	436 (28.9)	64 (14.7)	0.50	(0.39, 0.63)	0.60	(0.46, 0.78)^g^
No	1,072 (71.0)	317 (29.6)	Ref		Ref	
Missing	1 (0.1)	0 (0.0)	--		^–^	

Abbreviations: aRR, adjusted relative risk; CI, confidence interval; DM, diabetes mellitus; N/A, not available; RR, crude relative risk; SeWA, Severe TB Web Application.

a Aged 15 years and older.

b Defined as the presence of any 1 indicator during triaging: body mass index ≤14 kg/m^2^/14.1–16.0 with bilateral pedal edema, respiratory rate >24 breaths/minute, oxygen saturation <94%, not able to stand without support.

c Source is the routinely collected baseline data in NIKSHAY and data updated in TB SeWA as of July 29, 2022.

d Column percentage.

e Row percentage.

f Modified Poisson regression with robust variance estimation; results have been adjusted for district.

g *P*<0.05.

h Variables with crude *p* value ≥ 0.05 (Chi square test) were not included in the adjusted analysis.

i Body mass index ≤14 kg/m^2^/14.1–16.0 with bilateral pedal edema.

j Respiratory rate >24 breaths/minute or oxygen saturation <94%.

## DISCUSSION

To the best of our knowledge, this is the first published experience of the description and predictors of losses in a statewide differentiated TB care cascade from routine program settings. We did not find similar studies for comparison that implemented the differentiated TB care cascade. We discuss key operationally relevant findings and recommendations.

We believe this to be the first published experience of the description and predictors of losses in a statewide differentiated TB care cascade from routine program settings.

### Burden of High Risk of Severe Illness

The burden of high risk of severe illness, though of public health importance, was lower than 35% in the neighboring state of Karnataka (October–November 2020) and 42% in Gujarat, western India (June 2021).^8^^,^^9^ The proportion of severe undernutrition among adults with TB in Tamil Nadu (25%) was lower than in Karnataka (30%) and Gujarat (38%).^8^^,^^9^ This is possibly a reflection of lower undernutrition levels among adults in the general population in Tamil Nadu (12%–13%) when compared to Karnataka (14%–17%), Gujarat (21%–25%), and national figures (16%–19%).^27^ We may also attribute the higher burden of high risk of severe illness in Karnataka and Gujarat to the preceding first and second COVID-19 wave, respectively.^8^^,^^9^

Very severe undernutrition was observed in 6% of people with TB and severe undernutrition in 25%. As a result of TN-KET, Tamil Nadu now routinely captures BMI data for all adults with TB (notified from public facilities). In addition to the monthly direct benefit transfer of 500 Indian rupees per month to all people with TB, the state now has the required information to consider targeted nutritional supplementation for those with BMI<16 at diagnosis.

Half of the patients with high risk of severe illness had very severe undernutrition. This implies the need for capacity-building of TN-KET nodal inpatient care facilities in clinical management of very severe undernutrition in adults, in line with the guidelines in the *Inpatient Care Guide for Adults With TB Who Are Severely Ill*.^25^ This management includes targeting admission duration longer than 7 days for patients with very severe undernutrition also. During the first week of inpatient care for these patients, the focus is on patient stabilization and initiation of cautious feeding followed by a few weeks of rehabilitation (duration of rehabilitation may depend on clinical condition and bed availability).^25^

### Feasibility of TN-KET and Its Implications

The first quarter of implementation suggests that TN-KET was feasible to implement, with scope for further improvement. Overall, 8 in 10 people were triaged; 8 in 10 people classified as high risk of severe illness were comprehensively assessed; and 9 in 10 confirmed severely ill people received inpatient care. There was minimal delay in tri-aging and more than half of the severely ill patients were admitted within 1 day of diagnosis. Data on all 5 triaging indicators were collected for most people. Following its successful implementation without additional stress or strain on the health system, we believe TN-KET to be a sustainable care model.

TN-KET was successfully implemented without additional stress or strain on the health system.

Two quality-related issues should be addressed. First, 1 in 10 people with high risk of severe illness were not confirmed as severely ill after comprehensive assessment at nodal TN-KET inpatient care facility (our target is less than 1 in 20). We were unable to objectively assess the reasons for this, as TB SeWA did not capture details of comprehensive assessment.

Most of the patients with high risk of severe illness, if comprehensively assessed, would have been confirmed as severely ill, unless there was an error in measuring the indicators during triaging. This being operational research, we cannot rule out measurement and recording errors. Beyond confirmation, the utility of comprehensive assessment is to identify the reasons for severe illness (that need to be addressed during inpatient care) and decide the level of inpatient care (secondary or tertiary). Differences in measurement could also be attributed to BMI having a “<” sign in the criteria for confirmation of severe illness while the triaging tool used a “≤” sign (Table 1 and Box).

Starting August 2022, we introduced a case record form for standardized comprehensive assessment in all TN-KET inpatient care facilities. In TB SeWA, we have included 2 new variables: (1) total score after comprehensive assessment and (2) reason for nonconfirmation of severe illness among referred adults with high risk of severe illness (open-ended). These will help us understand better why an adult with high risk of severe illness was not confirmed despite comprehensive assessment.

The second quality issue is that the median admission duration among severely ill people was only 4 days (our target is 7 days). This requires infrastructure strengthening in the nodal inpatient care facilities, including F-75/100 for management of very severe undernutrition, high protein diet, infection prevention and control, and many others.^19^^,^^20^^,^^25^

### Predictors of Losses in TN-KET Care Cascade

To improve retention in care cascade, we should act on predictors of not being triaged and comprehensively assessed. To improve coverage of triaging further, we intend to focus on districts with high numbers of notifications, patients with extrapulmonary TB (by resensitizing staff on who should be triaged), patients diagnosed at tertiary level facilities (by taking steps to ensure better coordination for triaging at diagnosis among various departments in the teaching hospitals), and those transferred out of the district (by ensuring triaging at diagnosis, especially at higher-level facilities).

To increase comprehensive assessment, we need to reinforce that all adults with high risk of severe illness are eligible. People with the ability to stand without support were less likely to be prioritized for comprehensive assessment, which may be attributed to nonreferral or refusal at nodal inpatient facilities. To ensure that eligible patients from primary and secondary level facilities are referred for comprehensive assessment, we need to ensure that the nodal TN-KET inpatient care facilities at secondary level—possibly closer to where patients live—also admit patients. Anecdotal experience suggests most of the admissions are happening at the tertiary level (i.e., teaching hospitals). We are not sure why being aged 25–34 years (when compared to aged 35–44 years) is a predictor for not being comprehensively assessed, as this study only quantitatively assessed the predictors. The answers to “why” will be available through qualitative systematic enquiry, which is ongoing.

### Way Forward

Effective implementation of the recommendations will help achieve the targets of 80-80-80-7 for the monitoring indicators at both state and district levels. By paying specific attention to the predictors of losses in the care cascade, the state has already achieved 90-87-95-6 for its indicators in the October–December 2022 quarter. This achievement combined with quality inpatient care will improve surrogate indicators being tracked by the state and districts, such as (1) median time to death (increase), (2) home deaths (decrease), and (3) early deaths (decrease). At the state level, among deaths during July 2022, the median time to death was 38 days as compared to 18 days during March 2022. The percentage of home deaths has decreased from 59% in March 2022 to 42% in July 2022. The percentage of early deaths remains the same at 70%–75%.

By paying specific attention to the predictors of losses in the care cascade, the state has surpassed most of its monitoring targets by the end of 2022.

Once there is an improvement in surrogate indicators, we may expect a decrease in TB deaths. Based on what we observe and feasibility, we may introduce the following: (1) systematic follow-up of patients with high risk of severe illness at 1 month and 2 months using the same triaging tool and/or (2) addition of systematic triaging of all TB patients at 2 months.

A qualitative systematic enquiry is also ongoing to explore the enablers, barriers, and suggested solutions as perceived by patients and providers. We plan to hold a review/audit of the quality of comprehensive assessment and inpatient care.

While we do this, we also understand that to reduce TB deaths we need to strengthen existing program activities and implement additional interventions beyond TN-KET. This includes addressing the social determinants and improving case finding so that patients are diagnosed and initiated on treatment early. To monitor ongoing case-finding strategies, 2 indicators are available for monitoring: (1) the trend of proportion diagnosed from primary level facilities (16% in April–June 2022) and (2) the trend of proportion of adults with TB with high risk of severe illness at diagnosis (now available as a result of TN-KET).

## CONCLUSION

With the goal of reducing TB deaths, the state of Tamil Nadu, India, prioritized TB patients with very severe undernutrition, respiratory insufficiency, or poor performance status at diagnosis for comprehensive assessment and inpatient care. This operational research aimed to determine the burden of patients with these conditions at diagnosis, losses and delay in the care cascade, and predictors of the losses. The burden of patients eligible for referral for comprehensive assessment (13%) was lower than reported in Karnataka and Gujarat, and half of those eligible had very severe undernutrition. The care cascade indicators and time intervals were satisfactory except for the admission duration.

With further reduction of losses in the care cascade during the October–December 2022 quarter, we intend to focus on ensuring the sufficient duration and quality of inpatient care. We hope that this will translate into a reduction in home deaths and early deaths in the short term and the reduction in the overall mortality rate over the long term in Tamil Nadu and ultimately contribute toward the national goal of achieving the World Health Organization End TB targets for 2030 by 2025.^1^^,^^2^

## Supplementary Material

GHSP-D-22-00505-supplement1.pdf

GHSP-D-22-00505-supplement3.xlsx

GHSP-D-22-00505-supplement2.pdf

## Notes

**Peer Reviewed**

**First published online:** April 3, 2023.
