# Supplementary material for: The First Differentiated TB Care Model From India: Delays and Predictors of Losses in the Care Cascade
Source: Glob Health Sci Pract. 2023 Apr 28;11(2):e2200505. doi: 10.9745/GHSP-D-22-00505 (PMC10141439; doi:10.9745/GHSP-D-22-00505)
Supplement: GHSP-D-22-00505-supplement1.pdf [file GHSP-D-22-00505-supplement1.pdf]

Supplement to: Shewade HD, Frederick A, Giruthika G, et al. The first differentiated TB care model from India: delays and predictors of losses in the care cascade. *Glob Health Sci Pract.* 2023;11(2):e2200505. https://doi.org/10.9745/GHSP-D-22-00505

Tamil Nadu *Kasanoi Erappila Thittam* (TN-KET)
Screening for severe illness at notification - Paper-based data collection tool (Single paper, Front n Back Print)

Starting 01April 2022, as a routine, all ADULTS (15 completed years or above) with TB (not known to be drug resistant at diagnosis) notified from public PHIs should be screened. Screening must be done as early as possible at diagnosis (without having to wait for the notification to happen) or at the next earliest opportunity (home visit or treatment start or baseline investigations of TB patient at PHI). If 'high risk of severe illness' is present (yes), please mark it in the notification register at 'Diagnosis' PHI and immediately inform the MO and STS for local level action (referral and inpatient care). Store these filled forms in a file at 'Diagnosis' PHI. **A copy of this form may be stapled with the TB treatment card**. These details will then be filled by the 'Diagnosis' TU STS in the Severe TB Web Application (TB SeWA). This is being implemented in NTEP districts of Tamil Nadu

Nikshay ID and date of notification (dd/mm/yyyy): _______________ and ______________________

NTEP 'Diagnosis' District: ______________

'Diagnosis' TU name: ____

'Diagnosis' Public Peripheral Health Institution (PHI) name: ____________________

Is the TB patient admitted in hospital at the time of screening (Tick one option)

○ No ○ Yes

- Weight (kilograms (kg), rounded to one decimal point - compulsory) - ___________________
- Height (centimetre, no decimal point) – ___________________
- BMI (kilogram per metre square (kg/m^2), one decimal point, compulsory) - _________________
  (Use only N-TB Application for calculating BMI)
- Swelling in the leg (press for 15 sec inner side, 2cm above the ankle), tick only one option
  ○ No ○ Yes
- Respiratory rate per minute (in sitting position after 30 min rest) - ___________
- Oxygen saturation (%) using pulse oximeter measured after 30 min rest- ___________
- Able to stand without support (tick appropriate response), choose only one option
  ○ No (not able to stand without support) ○ Yes (standing without support)
- **High risk of severe illness,** tick only one option (see criterion on the back side of this page)
  ○ No ○ Yes

Date of completing screening and this form at PHI (dd/mm/yyyy): _______________

Name and designation of staff completing the form: ____

Name, Signature (Seal) of PHI Medical Officer:

(All details filled in this form are correct and have been cross-checked by me)

If 'high risk of severe illness' is yes, then the following to be filled here and updated in TB SeWA

(Circle the correct option)

Whether referred for comprehensive assessment? ○ No ○ Yes, **If yes,** date (dd/mm/yyyy) of referral: ___________

Whether comprehensive assessment done? ○ No ○ Yes

Whether confirmed as severely ill? ○ No ○ Yes

Admitted for inpatient care? ○ No ○ Yes, **If yes**, date (dd/mm/yyyy) of admission: _________

Supplement to: Shewade HD, Frederick A, Giruthika G, et al. The first differentiated TB care model from India: delays and predictors of losses in the care cascade. Glob Health Sci Pract. 2023;11(2):e2200505.
https://doi.org/10.9745/GHSP-D-22-00505

Tamil Nadu *Kasanoi Erappila Thittam* (TN-KET)

FOR INFORMATION ONLY – Screening tool for severe illness

If at least one of the following is present (any one or more), then the adult with TB is

'high risk of severe illness - yes'

If all of the following are absent, then the adult with TB is

'high risk of severe illness - no'

1. Body mass index (BMI) less than or equal to (≤) 14.0 kg/m^2
2. BMI 14.1 to 16.0 kg/m^2 with leg swelling
3. Respiratory rate more than (>) 24 per minute
4. Oxygen saturation less than (<) 94%
5. Not able to stand without support (standing with support / squatting / sitting / bed ridden)
