# Supplementary material for: The First Differentiated TB Care Model From India: Delays and Predictors of Losses in the Care Cascade
Source: Glob Health Sci Pract. 2023 Apr 28;11(2):e2200505. doi: 10.9745/GHSP-D-22-00505 (PMC10141439; doi:10.9745/GHSP-D-22-00505)
Supplement: GHSP-D-22-00505-supplement2.pdf [file GHSP-D-22-00505-supplement2.pdf]

Supplement to: Shewade HD, Frederick A, Giruthika G, et al. The first differentiated TB care model from India: delays and predictors of losses in the care cascade. *Glob Health Sci Pract*. 2023;11(2):e2200505. https://doi.org/10.9745/GHSP-D-22-00505

Tamil Nadu *Kasanoi Erappila Thittam* (TN-KET)

Implementation of Systematic Screening, Referral and Inpatient Care for Severely Ill People with Tuberculosis in Tamil Nadu: a Differentiated Care Model Targeted towards Ending TB Deaths

STANDARD OPERATING PROCEDURE

Purpose: The purpose of this SOP is to outline the procedure to be followed in TN-KET

Responsibility: At 'Diagnosis' public PHI, with the support of laboratory technician and TB-health visitor (if present), the nodal person identified for notifying TB patients, should fill the paper-based data collection tool. Please get the paper-based data collection tool reviewed by the PHI Medical Officer. At 'Diagnosis' TB unit level, STS should transcribe the data from paper-based data collection tool to Severe TB Web Application (**TB SeWA**). Paper-based data collection tool should be filled first and then the data should be transcribed into the TB SeWA. District TB Officer (DDTB/DTO) and District Programme Coordinator (DPC) should monitor the indicators of TN-KET (shared later) at district level.

Summary: Starting 01April 2022, as a routine, all ADULTS (15 completed years or above) with TB (not known to be drug resistant at diagnosis) notified from public PHIs should be screened. Those with high risk of severe illness (yes) should be referred to TN-KET nodal inpatient care facility in the district. Screening must be done as early as possible at diagnosis (without having to wait for the notification to happen) or at the next earliest opportunity (home visit or treatment start or baseline investigations of TB patient at PHI). If 'high risk of severe illness' is present (yes), please mark it in the notification register at 'Diagnosis' PHI and immediately inform the MO and STS for local level action (referral and inpatient care). Store these filled forms in a file at 'Diagnosis' PHI. **A copy of this form may be stapled with the TB treatment card**. These details will then be filled by the 'Diagnosis' TU STS in the Severe TB Web Application (TB SeWA). Update the additional questions for those with high risk of severe illness (yes) as and when information is available (in paper-based screening tool at Diagnosis PHI and in TB SeWA by STS of Diagnosis TB unit). This is being implemented in NTEP districts of Tamil Nadu.

The purpose of this activity is not data collection or data dissemination but to reduce TB deaths. The key is to use this information for local level action to reduce TB deaths. Local level means PHI level first, TB unit level if PHI MISSED taking local action. Local level action includes referring adults with 'high risk of severe illness (yes)' to TN-KET nodal inpatient care facility in the district for comprehensive assessment, confirmation of severe illness, identifying the issues to be addressed and inpatient care. Indicators to monitor TN-KET have been provided.

Supplement to: Shewade HD, Frederick A, Giruthika G, et al. The first differentiated TB care model from India: delays and predictors of losses in the care cascade. Glob Health Sci Pract. 2023;11(2):e2200505. https://doi.org/10.9745/GHSP-D-22-00505

NIKSHAY ID and DATE OF NOTIFICATION

- The Episode ID for the current TB episode will be the NIKSHAY ID
- The date of diagnosis (date of testing) for the current TB episode that is reflected in NIKSHAY will be considered as the date of notification

DATE FORMAT

- Date format is dd/mm/yyyy, for ex, 26/05/2022 is the correct date format.
- 26 May 2022, 26/5/22 or 26/5/2022 are wrong date formats.

WEIGHT:

- Weight to be recorded after removing heavy warm clothing, belt and shoes
- Use the available weighing machine.
- The participant is be made to stand still and upright with weight evenly distributed between the two feet.
- Remember to ask the participant to not look down to check his or her own reading so as to measure correct reading.
- Take 2 readings if needed and enter the average in the paper based data collection tool (weight in kg, rounded to one decimal point).
- Record the weight in kg, rounded off to one decimal point (at least one decimal point compulsory (Example 67.0 kg, 70.3 kg, 45.5 kg)

HEIGHT:

- Height to be measured using a portable stadiometer (be kept on an even surface) or any other standard mechanism used in the PHI to measure height.
- The participant to be instructed for the following:
- Remove foot wear.
- Remove buns and headgears that interfere with measurement.
- Ensure contact of 4 points: head, shoulder blade, buttocks and heels.
- Weight to be borne evenly on both feet, heels almost together with feet pointing outwards at 60 degree angle.
- Rest the Stadiometer headpiece on the head to just compress the hair.
- Ask the person to take a deep breath and hold it: this straightens the spine and makes the measurement reproducible.
- If the participant is taller than the examiner, use a stool.
- Record the readings in the paper-based data collection tool (height in cm, no decimal point)

BODY MASS INDEX (BMI):

- **Install the N-TB Mobile Application (download from playstore) in the mobile / smart phone. Use N-TB Mobile Application to calculate BMI in kg/m^2. We do not encourage manual calculation, as these are prone for errors.**
- Enter the height in centimetre (no decimal point)
- Enter the weight in kg (rounded to one decimal point)
- N-TB App will give the BMI in kg/m^2 with one decimal point. Enter the BMI in paper based data collection tool (included one decimal point, example 20.8 kg/ m^2, 26.0 kg/ m^2, 17.3 kg/ m^2). If the N-TB App does not mention a decimal point, then add '.0'. For example, if BMI is 26 kg/ m^2 in N-TB App, mention it as 26.0 in paper-based data collection tool. If it is 14 kg/ m^2 , in N-TB App, mention it as 14.0 in paper-based data collection tool

SWELLING OF LIMBS

- Expose the patient's legs and examine them with the patient standing/lying supine.
- Press gently with your fingertip over the bony prominence 2cm above the ankle for a 15 seconds and then see if your finger has left a pit.
- Remember to avoid areas with wounds.

RESPIRATORY RATE:

- This should be measured after rest of at least 30 minutes
- Quietly observe the participant's breathing pattern without drawing their attention to it, as this may cause it to change.
- Sleeping position may be considered for easy count
- Count the number of times the participant inhales (inhalation and exhalation is one full count) for a full one minute.
- Record the number of breaths per minute in the paper based data collection tool.

PULSE OXIMETRY

This should be measured after rest of at least 30 minutes

A clip-like device called a probe is placed on the index finger. The probe uses light to measure how much oxygen is there in the blood.

- Normal should be 94% and above

- If less than 94% please check on the fingers of your own hand and the other hand of the patient
- If still less than 94% the patient must be assessed by the medical officer

If less than 90, consider it as a medical emergency and facilitate admission of the patient in the nearest government hospital. After recording enter the reading in the paper based tool.

STUDY PHASES

- Jan-Mar 2022 is the preparatory period.
- 14-27 March 2022 is the period to implement the pilot in all the 30 NTEP districts.
- Apr 2022 onwards, TN-KET will be implemented systematically as a routine activity in the programme.

OPERATIONAL DEFINITION OF STUDY POPULATION

Study population (patients who have to undergo screening for severe illness)

- Study population is ALL adults (15 completed years and above) notified with TB from public PHIs of the 30 study districts of Tamil Nadu (except Chennai), starting 01 April 2022 (date of notification) and not known to be drug-resistant at diagnosis.
- Though we say that adults notified from 01 Apr 2022 form the study population, please note that we will do a pilot exercise during a two week period in March 2022 (study population notified between 14 and 27 March 2022).
- Adults is defined as age in completed years more than or equal to 15 years. This includes 15 completed years, 16 completed years and so on. This does not include 14 completed years, 13 completed years and so on. 14 years and 11 months is 14 completed years so the person is not adult.
- Among adults with TB notified from public PHIs, if they undergo any of the WHO recommended rapid diagnostic tests as a part of diagnosis (test of diagnosis), then we may have information on resistance at diagnosis.
- There could be patients who are known to be drug-sensitive at diagnosis (if WHO recommended rapid diagnostic tests is the test of diagnosis). These have to be included.
- There could be patients whose drug-resistance status is unknown at diagnosis (eg if sputum microscopy is the test of diagnosis). These have to be included irrespective of the drug-resistance status that is detected later on during the universal DST pathway.
- At diagnosis, if resistance is detected to any one anti-TB drug, then the patient is someone with ‘known drug-resistance at diagnoses’. These patients get referred for DR-TB care and should be excluded from study population.

Supplement to: Shewade HD, Frederick A, Giruthika G, et al. The first differentiated TB care model from India: delays and predictors of losses in the care cascade. Glob Health Sci Pract. 2023;11(2):e2200505.
https://doi.org/10.9745/GHSP-D-22-00505

SCREENING, REFERRAL AND FILLING OF DATA

- The purpose of screening is to screen as soon as possible after diagnosis or notification. Most of the deaths happen in the weeks following diagnosis and hence, any delay in screening means missing the opportunity to prevent a TB death.
- **The paper-based data collection tool is a 'two page, single paper' tool. The printing should be done front and back to ensure that it is a 'two page, single paper tool'.**
- As and when a patient is detected as 'high risk of severe illness – yes', this should also be denoted (say with a red tick) in the 'Diagnosis' PHI level notification register.
- As and when a patient is detected with 'high risk of severe illness – yes', the PHI should intimate the STS and MOTC for needful local action.
- Screening must be done as early as possible (at diagnosis, without having to wait for the notification to happen) or at the next earliest opportunity (home visit or treatment start or baseline investigations of TB patient at PHI).
- The PHI should try to fill the paper-based data collection tool as soon as a patient is diagnosed (on the same day). NIKSHAY ID can be filled later (as and when it is available) in the paper-based data collection tool. The reason being, for district hospitals, medical colleges, the day of diagnosis may be the only opportunity for severity screening. The patient may get transferred out and the opportunity for screening may be lost if we wait for generation of NIKSHAY ID. Severity screening must be done for all study populations irrespective of the treatment initiation status or transfer out status. If transferred out immediately after diagnosis/notification and the patient was not screened, the patient should be followed up and severity data should be requested from the PHI where the patient has been transferred. It is the responsibility of the PHI of notification to maintain the paper-based data of severity screening.
- The filled paper-based data collection tools should be maintained in a file at 'Diagnosis' PHI level. A copy of this should be stapled with TB treatment card.
- As soon as possible, the STS should transcribe the severity screening data from paper-based data collection tool to TB SeWA. Before transcribing, the data should be checked, especially the interpretation of 'high risk of severe illness' status. If there is any error, the PHI should be asked to make corrections in the paper-based data collection tool.
- While transcribing data in the TB SeWA, BMI and 'high risk of severe illness' are auto generated fields. Once data is transcribed in TB SeWA, if there is any discrepancy in paper-based data collection tool and TB SeWA data, then the corrections should be reflected in both the sources.
- **Transcribing of data into TB SeWA should be done only by the STS.** On behalf of STS, the DPC or higher level supervisors should NOT transcribe data in the TB SeWA; they should monitor the data collection.
- As and when a patient is detected as 'high risk of severe illness – yes', this should also be denoted (say with a red tick) in the 'Diagnosis' TB Unit level notification register (like it is done at 'Diagnosis' PHI level).

- Many patients gets diagnosed /notified at district level hospital and then get transferred to rural /urban primary health centre for treatment. These patients should be screened for severe illness before transferring to primary health centre for treatment (mechanisms to be worked out to deal with the burden of cases). There is no point in transferring to primary health centre, then tracking patient to the primary health centre and asking the primary health centre to screen for severe illness, if found to be severe, again asking to send the patient from PHC to district hospital. The need for screening at diagnosis at 'diagnosing PHI' is best exemplified here.

- **In addition to screening related data, we have to systematically fill referral related data** [for those with high risk of severe illness (yes)] in the paper-based screening tool (last four questions at the bottom). This should be filled by the same PHI level nodal staff that was involved in filling screening related data. Similarly, the STS in TB SeWA, as and when available, should also transcribe this information. The DPC/DTO has access to the online monitoring tool which has details of district level nodal inpatient care facilities along with the contact details of medical / paramedical nodal persons in these facilities. Local mechanisms may be made so that this information is shared at block level and PHI level. This will aid in tracking of referred patients.

- **Filling of paper-based screening tool (by PHI nodal person) and TB SeWA (by STS) may therefore be done in two settings**. First setting is when screening related data is filled. Second, setting is when, for those with high risk of severe illness (yes), referral and post-referral data is available and ready to be filled.

MONITORING OF SCREENING FOR SEVERE ILLNESS AND REFERRAL

- Monitoring should be done on a weekly basis by the district programme coordinator (DPC) and indicated in the online monitoring tool (Excel spread sheet shared over Google Drive). This tool has three sheets: the first one has district wise details, the second sheet in the monitoring tool while the third sheet contains the Gantt chart. In the Gantt Chart you will find the Jan-Mar 2022 is the preparatory period, March is for pilot and starting April 2022, TN-KET will be implemented. DPC have been provided access to the online monitoring tool and it is their responsibility to update information. For example converting 'no' to 'yes' as applicable and ensure the district level and TB Unit level contact details and data are correct and updated.
- Specific days when monitoring should be done and for the period for which it should be done has been mentioned in the online monitoring tool.
- Monitoring of screening means cross checking the completeness and correctness of data on a specific day for a specific period.
- Completeness of screening includes reviewing the cumulative total number (since Apr 01, 2022) of adult public notified TB patients not known to be drug-resistant (study population-denominator) and total number of screening for severe illness done (numerator). The source of the denominator is NIKSHAY and the source of the numerator is TB SeWA. While downloading the denominator for the district from NIKSHAY (using following filters range of date of notification, public notified,

drug-sensitive TB and age≥15y), the DPC should use the 'diagnosed/notified' option, instead of the 'current' option. If 'current' option is used, the adults who are lost to follow up, have died or transferred out (as on the date of download) will not appear in the cumulative numbers, giving a false inflated completeness of screening.

- **In addition to comparing aggregate numbers, we recommend that STS and DPC maintain a line list of NIKSAY IDs of study population from the start of 01 April 2022, from earliest to latest (in an Excel sheet).** They may colour code the cells using green as and when screening gets completed. If NIKSAY ID of a person who fits into the study population is available in the web portal of the TB SeWA (irrespective of the correctness of data and extent of missing data) that means screening has been done (screening –yes). If NIKSAY ID of a person who fits into the study population is NOT available in the web portal of the TB SeWA that means screening has NOT been done (screening – no). It is possible that the PHI staff may screen the prevalent TB patients (notified before 01 Apr 2022, not part of study population) and this may again give a false inflated completeness of screening.
- Correctness includes, of those screened, the data collected does not have errors especially the interpretation of severe illness.
- Has appropriate action been taken after cross-checking of completeness and correctness? Once completeness, correctness has been assessed and appropriate action has been taken by the STS (based on DPC feedback), this means DPC has cross checked the monitoring data for screening.
- Similarly, the DPC should monitor the referral data. Of those with high risk of severe illness (yes), how many referred, how many completed comprehensive assessment, how many many confirmed as as severely ill and how many were admitted for inpatient care. Has appropriate action been taken after cross-checking of referral related data. Once, this is done, it may be denoted as 'yes' in the online monitoring tool.
- **TN-KET Monitoring indicators (source TB SeWA – View Screening Data - Reports)**
    - % Screened (Yes/No) among adult public notified drug sensitive patients at diagnosis (source of denominator is NIKSHAY – discussed before)
    - % High risk of severe illness (yes / no / unknown) among adult public notified drug sensitive patients at diagnosis (source of denominator is NIKSHAY – discussed before)
    - Of those with high risk of severe illness - yes
        - % Referred (Yes/No)
        - % Comprehensive Clinical Assessment (Yes/No)
        - % Confirmed as severely ill (Yes/No)
        - % Inpatient care (Yes/No/NA)
- As NIKSHAY ID will be used to match the NIKSHAY and TB SeWA data, the 'Diagnosis' PHI staff and 'Diagnosis' PHI STS should be very diligent while entering this NIKSAY ID (avoid errors) in paper-based data collection tool and TB SeWA, respectively.

IMPORTANT DATES

- **11 Mar 2022** – the deadline by when all the districts should be prepared and ready to implement TN-KET. This should be updated in the online monitoring tool by the DPCs.
- 14 Mar 2022 – start of pilot
- 01 April 2022 – start of TN-KET
